# Effects of dexmedetomidine on surgery for type A acute aortic dissection outcome

**DOI:** 10.1038/s41598-022-06710-w

**Published:** 2022-02-17

**Authors:** Yu-Ting Cheng, Kuang-Tso Lee, Chih-Hsiang Chang, Victor Chien-Chia Wu, Yi-Shin Chan, Dong-Yi Chen, Pao-Hsien Chu, An-Hsun Chou, Kuo-Sheng Liu, Shao-Wei Chen

**Affiliations:** 1 Division of Thoracic and Cardiovascular Surgery, Department of Surgery, Chang Gung Memorial Hospital, Linkou Medical Center, Chang Gung University, Taoyuan City, Taiwan; 2 Department of Cardiology, Chang Gung Memorial Hospital, Linkou Medical Center, Guishan District, Chang Gung University, No. 5 Fuxing Street, Taoyuan City, 33305 Taiwan; 3 Department of Nephrology, Chang Gung Memorial Hospital, Linkou Medical Center, Chang Gung University, Taoyuan City, Taiwan; 4 Department of Nephrology, Kidney Research Center, Chang Gung Memorial Hospital, Taoyuan City, Taiwan; 5 Department of Anesthesiology, Chang Gung Memorial Hospital, Linkou Medical Center, Chang Gung University, Taoyuan City, Taiwan; 6 Center for Big Data Analytics and Statistics, Chang Gung Memorial Hospital, Linkou Medical Center, Taoyuan City, Taiwan

**Keywords:** Acute kidney injury, Aortic diseases

## Abstract

No study has evaluated the effect of dexmedetomidine in patients who received surgery for type A aortic dissection. This is the first study to evaluate the effect of dexmedetomidine in aortic dissection patients. This study was executed using data from the Chang Gung Research Database in Taiwan. The CGRD contains the multi‐institutional standardized electronic medical records from seven Chang Gung Memorial hospitals, the largest medical system in Taiwan. We retrospectively evaluate patients who received surgery for acute type A aortic dissection between January 2014 and December 2018. Overall, 511 patients were included, of whom 104 has received dexmedetomidine infusion in the postoperative period. One-to-two propensity score-matching yielded 86 cases in the dexmedetomidine group and 158 cases in the non-dexmedetomidine group. The in-hospital mortality and composite outcome including all-cause mortality, acute kidney injury, delirium, postoperative atrial fibrillation, and respiratory failure, were considered primary outcomes. The in-hospital mortality and composite outcome were similar between groups. The risk of Acute Kidney Injury Network stage 3 acute kidney injury was significantly lower in the dexmedetomidine group than in the non-dexmedetomidine group (8.1% vs 19.0%; OR, 0.38; 95% CI, 0.17–0.86; *p* = 0.020. The risk of newly-onset dialysis was also significantly lower in the dexmedetomidine group than in the non-dexmedetomidine group (4.7% vs 13.3%; OR, 0.32; 95% CI, 0.11–0.90; *p* = 0.031). Post-operative dexmedetomidine infusion significantly reduced the rate of severe acute kidney injury and newly-onset dialysis in patients who received surgery for acute type A aortic dissection.

## Introduction

Acute type A aortic dissection is a life-threatening true surgical emergency. Despite aggressive operation and technique advance, surgical mortality is still high. According to The German Registry for Acute Aortic Dissection Type A (GERAADA), from 2006 to 2010, 2137 patients have surgically treated for acute type A aortic dissection with an overall 30-day mortality of 16.9%^1^. Another report from the International Registry of Acute Aortic Dissection (IRAD) shows, over time, from 1995 to 2013, the in-hospital surgical mortality rate of patients with type A aortic dissection dropped significantly from 31%, but still as high as 22%^2^. Acute type A aortic dissection also accompanies several major complications, including shock, tamponade, visceral ischemia, peripheral ischemia, and brain injury. Besides, minor complications including acute kidney injury, delirium, atrial fibrillation, and respiratory failure also occur commonly after the operation. Cardiopulmonary bypass (CPB) causes inflammation throughout the body, leading to organ dysfunction^3^. Research also showed an inflammatory mechanism is involved in medial degeneration and its association with the clinical manifestations of aortic dissection^4^.

Dexmedetomidine is a central α-2 adrenergic agonist, which has been shown to have anti-inflammatory properties, decreasing mortality and attenuating plasma cytokine concentrations in laboratory animals^5^. Dexmedetomidine infusion reveals anti-inflammatory effects during and after CPB. These suppressive effects of dexmedetomidine might be due to the inhibition of nuclear factor kappa B activation and suggest that intra-operative dexmedetomidine may beneficially inhibit inflammatory responses associated with ischemia–reperfusion injury during cardiopulmonary bypass^6^. A meta-analysis published in 2015 shows that perioperative use of dexmedetomidine as an adjunct to general anesthesia leads to significant decreases in serum levels of IL-6, IL-8, and TNF-α within a period of 24 h postoperatively^7^.

We hypothesized that dexmedetomidine may provide cardiac, brain, pulmonary, and renal protection for acute type A aortic dissection patients. This study aimed to investigate the effect of dexmedetomidine on the outcome of acute type A aortic dissection. In addition to investigating the endpoints of death, this study also examined the potential impact of dexmedetomidine on other major endpoints such as stroke, respiratory failure, fasciotomy or amputation, ECMO, postoperative infection, acute kidney injury, newly-onset dialysis, and acute respiratory distress syndrome during the postoperative period for patients undergoing surgery for acute type A aortic dissection.

## Methods

### Data source

This study was executed using data from the Chang Gung Research Database (CGRD) in Taiwan. The CGRD contains the multi‐institutional standardized electronic medical records (EMR) from Chang Gung Medical Foundation (CGMF) from seven Chang Gung Memorial hospitals (CGMH), including two medical centers, two regional hospitals, and three district hospitals from northern to southern Taiwan. The EMR contains the patient‐level data derived from electronic medical charts of patients established for administrative and health care purposes for CGMH. All the health care providers from CGMH can access the data for their clinical practice. The Department of Information Systems Management of CGMH integrated and standardized all EMR from CGMH without selecting criteria and established CGRD for research purposes. The basic architecture for the CGRD includes most of the information from EMR for routine epidemiologic health care studies, with nine profiles for laboratory data, inpatient data, outpatient data, emergency patient data, pathological data, nursing data, charge data, disease category data, and surgery data^8^. The CGRD has collected EMR of all patients from CGMH since 2000. Specifically, the health conditions are coded following the International Classification of Disease, 9th Revision Clinical Modification (ICD‐9‐CM) codes before 2016, and ICD‐10 codes afterward. The study was approved by the institutional review board (IRB) of Chang Gung Memorial Hospital and informed consent was waived. All research was performed in accordance with the Declaration of Helsinki concerning the ethical principles for medical research. All methods were carried out in accordance with relevant guidelines and regulations.

### Study population

**Figure 1:**
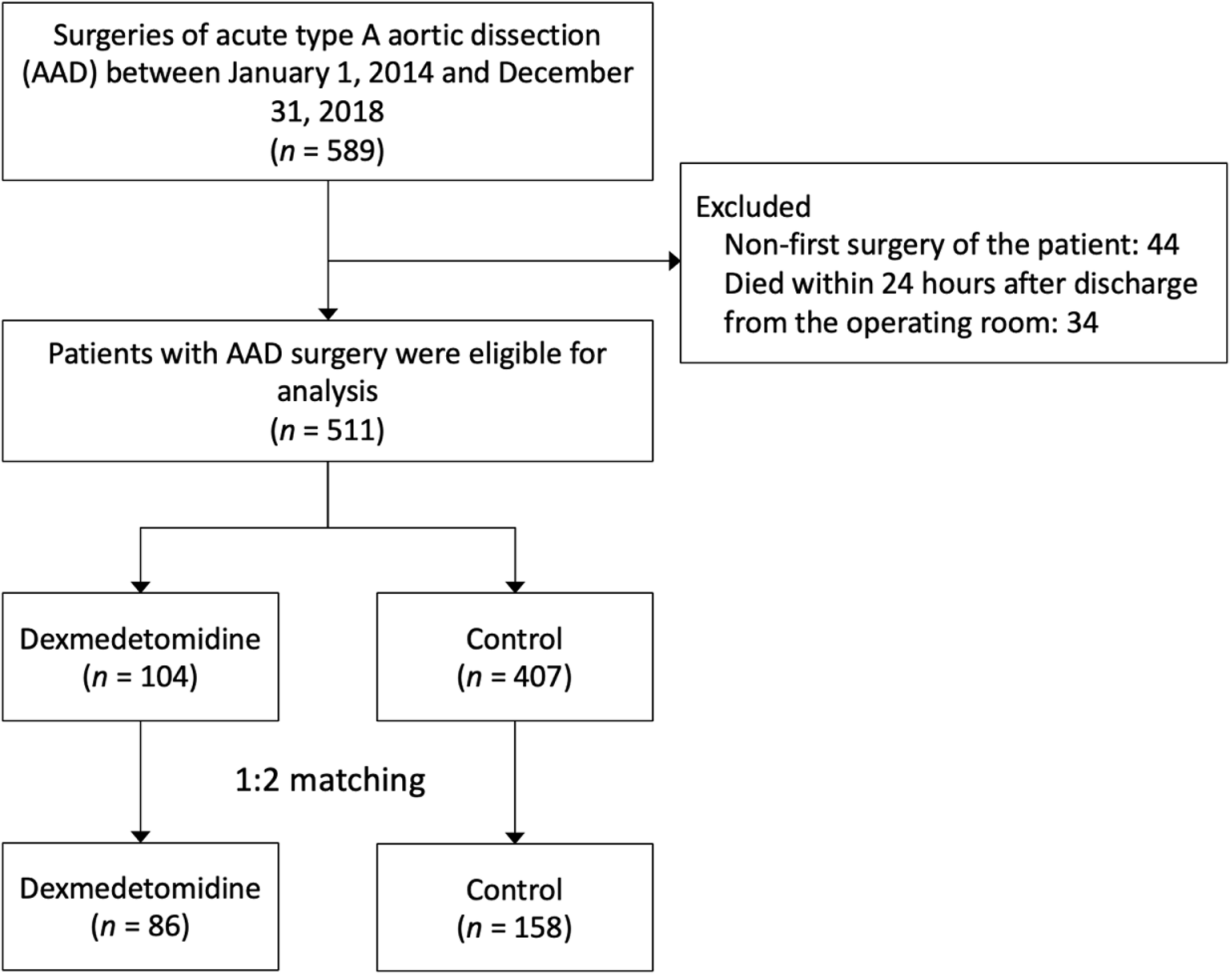
Inclusion of study patients.

The study cohort flowchart is shown in Fig. 1. This is a retrospective multi‐institutional cohort study. Patients hospitalized after surgery for acute type A aortic dissection between January 1, 2014, and December 31, 2018, were studied. Patients with a Taiwanese NHI procedure code for ascending aortic replacement (69,024, 68,043) were used for analysis. Patients who have duplicated surgical reports were excluded. The patient who has died within 24 h after operation were also excluded. All surgical reports were reviewed by two cardiac surgeons to make sure the enrolled patients were acute type A aortic dissection. The patients were then divided into two groups: the dexmedetomidine group and the non-dexmedetomidine group. The dexmedetomidine group was identified by using the medication supply code (BC24002212) in the post-operative periods for up to 24 h.

### Comorbidities and outcomes

Demographic data, clinical characteristics, and comorbidities were identified using ICD-9-CM or NHI procedure codes. Pre-operative lab data, post-operative lab, and surgical data were all derived from medical records. Perioperative outcomes were detected by tracing patients' hospitalization records. Outcomes of interest are the primary composite outcome and in-hospital mortality. Components of the primary composite outcome were all-cause mortality, acute kidney injury, delirium, postoperative atrial fibrillation, and respiratory failure.

The severity of acute kidney injury was categorized by Acute Kidney Injury Network (AKIN) criteria which was endorsed by the Kidney Disease Improving Global Outcomes (KDIGO) Clinical Practice Guideline. Hemodialysis was defined by the Taiwan NHI reimbursement code for hemodialysis or continuous veno-venous hemofiltration dialysis (58,001 or 58,018) Delirium was defined by positive CAM-ICU score evaluated by ICU nurse, use of haloperidol for agitated delirium, and confirmed by neurologist consultation. Postoperative atrial fibrillation was defined by reviewing of EKG report, using amiodarone for rhythm control, and reviewing discharge notes. Respiratory failure was identified using ICD-9-CM diagnostic codes. Patients were followed until death or December 31, 2018, whichever came first.

### Statistical analysis

Due to a substantial number of missing observations in the laboratory and surgical data, we first imputed the missing values using single expectation maximization (EM) imputation method and then created the propensity score matching (PSM) cohort. The PSM by multivariable logistic regression model was conducted to balance the baseline characteristics between the dexmedetomidine and non-dexmedetomidine groups. Each dexmedetomidine case was matched to two counterparts in the non-dexmedetomidine group if possible. The parameters used to calculate the propensity scores were age, sex, previous cardiac surgery, comorbidities, pre-operative condition, pre-operative lab data, surgical data, and surgical extension (listed in Table 1). The greedy nearest neighbor algorithm was adopted with a caliper width of 0.05 standard deviation of the logit of the propensity score. An absolute standardized difference (STD) of less than 0.2 after matching was considered to indicate a small difference between the groups. After matching, 72 and 14 patients in the dexmedetomidine had two and one counterparts respectively, resulting in a total of 86 patients with dexmedetomidine and 158 patients without dexmedetomidine.

**Table 1:** Baseline characteristics of the patients who received Dexmedetomidine versus who did not before and after propensity score matching.

		Before matching			After matching		
Variable	Valid *N*	Dexmedetomidine (*n* = 104)	Control (*n* = 407)	STD	Dexmedetomidine (*n* = 86)	Control (*n* = 158)	STD
**Demographics**							
Age, year*	511	56.4 ± 13.0	59.0 ± 13.3	− 0.20	56.9 ± 12.9	56.3 ± 13.8	0.05
Male sex*	511	70 (67.3)	274 (67.3)	< 0.01	57 (66.3)	105 (66.5)	< 0.01
BMI, kg/m^2^	472	31.1 ± 21.9	27.9 ± 16.5	0.17	28.3 ± 12.7	29.9 ± 24.7	− 0.08
Previous cardiac surgery*	511	1 (1.0)	9 (2.2)	− 0.10	1 (1.2)	2 (1.3)	− 0.01
**Comorbidities**							
Hypertension*	511	68 (65.4)	274 (67.3)	− 0.04	57 (66.3)	106 (67.1)	− 0.02
Coronary artery disease*	511	6 (5.8)	37 (9.1)	− 0.13	5 (5.8)	10 (6.3)	− 0.02
Marfan syndrome*	511	4 (3.8)	9 (2.2)	0.10	4 (4.7)	6 (3.8)	0.04
Diabetes mellitus*	511	6 (5.8)	39 (9.6)	− 0.14	5 (5.8)	8 (5.1)	0.03
Chronic kidney disease*	511	11 (10.6)	58 (14.3)	− 0.11	9 (10.5)	19 (12.0)	− 0.05
Liver disease*	511	12 (11.5)	48 (11.8)	− 0.01	11 (12.8)	11 (7.0)	0.20
Atrial fibrillation*	511	5 (4.8)	31 (7.6)	− 0.12	3 (3.5)	12 (7.6)	− 0.18
COPD*	511	5 (4.8)	18 (4.4)	0.02	4 (4.7)	2 (1.3)	0.20
Old stroke*	511	3 (2.9)	17 (4.2)	− 0.07	3 (3.5)	5 (3.2)	0.02
**Pre-operative conditions**							
Tamponade or shock*	511	8 (7.7)	64 (15.7)	− 0.25	8 (9.3)	13 (8.2)	0.04
Pre-operative lab data*							
Creatinine, mg/dL	494	1.33 ± 1.24	1.40 ± 1.31	− 0.06	1.33 ± 1.30	1.32 ± 1.13	0.01
WBC, 10^3^/uL	508	14.0 ± 5.2	13.1 ± 4.7	0.19	14.0 ± 5.2	13.6 ± 4.9	0.07
Platelet, 1000/uL	508	192.3 ± 81.4	180.1 ± 67.7	0.16	185.9 ± 52.1	192.1 ± 78.6	− 0.09
Hemoglobin, g/dL	508	13.5 ± 2.0	13.4 ± 2.0	0.08	13.5 ± 2.0	13.5 ± 1.8	− 0.01
BUN, mg/dL	372	19.6 ± 12.2	19.3 ± 9.5	0.03	18.8 ± 10.3	18.7 ± 8.8	0.01
Sodium, mg/dL	503	138.1 ± 3.8	138.8 ± 3.3	− 0.19	138.1 ± 3.7	138.4 ± 2.9	− 0.09
Potassium, mg/dL	503	3.8 ± 0.5	3.8 ± 0.6	0.07	3.8 ± 0.5	3.7 ± 0.5	0.09
Albumin, mg/dL	136	3.3 ± 0.5	3.3 ± 0.6	− 0.10	3.3 ± 0.4	3.4 ± 0.4	− 0.05
Lactic acid, mg/dL	138	51.6 ± 24.5	52.9 ± 35.8	− 0.04	53.1 ± 19.3	50.9 ± 22.7	0.10
HbA1c, %	163	6.3 ± 1.1	5.9 ± 0.5	0.36	6.0 ± 0.4	6.0 ± 0.3	< 0.01
AST, U/L	316	40.0 [25.0, 70.0]	38.0 [26.0, 65.0]	− 0.04	42.0 [26.0, 74.2]	40.5 [26.0, 74.0]	0.13
ALT, U/L	360	28.0 [20.0, 49.0]	25.0 [17.0, 40.0]	− 0.03	26.0 [18.0, 48.0]	22.6 [17.0, 33.0]	0.19
INR	499	1.15 ± 0.16	1.14 ± 0.18	0.10	1.16 ± 0.17	1.14 ± 0.21	0.10
**Post-operative lab data**							
Platelet, 1000/uL	508	145.9 ± 46.8	135.0 ± 43.3	0.24	145.1 ± 38.0	141.8 ± 46.2	0.08
Hemoglobin, g/dL	508	10.3 ± 1.5	10.7 ± 1.7	− 0.28	10.3 ± 1.5	10.7 ± 1.6	− 0.26
Lactic acid, mg/dL	269	56.5 ± 33.0	61.1 ± 40.9	− 0.12	58.8 ± 33.2	53.2 ± 33.0	0.17
Proteinuria, mg	155	30.0 [15.0, 100.0]	30.0 [15.0, 100.0]	− 0.10	42.5 [30.0, 76.6]	44.2 [30.0, 65.0]	0.05
AST, U/L	420	84.0 [54.0, 160.5]	85.5 [56.0, 176.5]	− 0.18	88.5 [54.0, 167.0]	74.7 [52.0, 179.4]	0.02
ALT, U/L	396	52.0 [32.0, 105.0]	48.0 [25.0, 107.0]	− 0.14	52.9 [34.0, 125.0]	48.0 [23.0, 130.9]	− 0.08
SOFA score	244	10.6 ± 2.2	10.9 ± 2.2	− 0.13	10.5 ± 1.9	10.8 ± 1.8	− 0.21
**Surgical data**							
Bypass time, min	385	241 ± 83	259 ± 68	− 0.25	250 ± 79	255 ± 65	− 0.07
Clamp time, min	391	155 ± 56	162 ± 49	− 0.13	159 ± 54	162 ± 46	− 0.07
Arrest time	261	37.7 ± 13.9	51.4 ± 25.9	− 0.66	46.1 ± 17.8	48.1 ± 20.5	− 0.11
**Brain protection**							
Antegrade	511	77 (74.0)	200 (49.1)	0.53	59 (68.6)	106 (67.1)	0.03
Retrograde	511	27 (26.0)	207 (50.9)	− 0.53	27 (31.4)	52 (32.9)	− 0.03
Cerebral perfusion, min	385	44.3 ± 19.6	52.0 ± 23.2	− 0.36	46.3 ± 17.9	48.1 ± 19.5	− 0.10
**Surgical extension**							
Partial or total aortic arch replacement	511	14 (13.5)	135 (33.2)	− 0.48	14 (16.3)	30 (19.0)	− 0.07
Aortic root replacement	511	3 (2.9)	15 (3.7)	− 0.04	3 (3.5)	6 (3.8)	− 0.02
Elephant trunk	511	7 (6.7)	28 (6.9)	− 0.01	7 (8.1)	13 (8.2)	< 0.01
Ascending aorta replacement only	511	80 (76.9)	232 (57.0)	0.43	62 (72.1)	109 (69.0)	0.07

STD, standardized difference; BMI, body mass index; COPD, chronic obstructive pulmonary disease; WBC, whole blood cell; BUN, blood urea nitrogen; HbA1c, glycated hemoglobin; AST, aspartate aminotransferase; ALT, alanine aminotransferase; SOFA, sequential organ failure assessment; INR, international normalized ratio;

* The covariates for propensity score matching;

Data were presented as frequency (percentage) or mean ± standard deviation or median [Quartile 1, Quartile 3].

Our statistical analysis methods have been described before^9^. In detail, the in-hospital outcomes between the dexmedetomidine and non-dexmedetomidine groups were compared using generalized estimating equation (GEE), in which the robust standard error was estimated to account for the outcome dependency within the same matched pair. When the link was identity, the distribution was normal for continuous outcomes. When the link was logit, the distribution was binomial for binary outcomes. The study group was the only explanatory variable in the aforementioned regression analyses. Any two-sided P-value that was less than 0.05 was considered to be statistically significant, and no adjustment of multiple testing (multiplicity) was made in this study. Statistical analyses were performed using SAS version 9.4 (SAS Institute, Cary, NC), including the procedures of “psmatch” for PSM and “genomd” for GEE.

## Results

### Study population characteristics

We identified 511 patients who received dexmedetomidine or non-dexmedetomidine after surgery for type A aortic dissection between January 1, 2014, and December 31, 2018. Table 1 lists the baseline characteristics, comorbidities, pre-operative condition, preoperative lab data, postoperative lab data, surgical data, and surgical extension in both study groups before and after PSM.

### In-hospital mortality and perioperative outcomes

Table 2 lists the in-hospital mortality and postoperative complication rates.

**Table 2:** Perioperative outcomes of the patients who received Dexmedetomidine versus who did not after propensity score matching.

Outcome	Dexmedetomidine (*n* = 86)	Control (*n* = 158)	*B*/ OR 95% CI	*P*
**Categorical parameter**				
Death within one month	5 (5.8)	22 (13.9)	0.39 (0.14, 1.07)	0.067
In-hospital mortality	5 (5.8)	22 (13.9)	0.39 (0.14, 1.07)	0.067
New onset ischemic stroke	11 (12.8)	22 (13.9)	0.91 (0.45, 1.84)	0.796
New onset hemorrhagic stroke	3 (3.5)	4 (2.5)	1.42 (0.38, 5.32)	0.602
Respiratory failure	5 (5.8)	16 (10.1)	0.55 (0.19, 1.62)	0.279
ECMO	5 (5.8)	14 (8.9)	0.63 (0.21, 1.88)	0.406
Postoperative infection	3 (3.5)	11 (7.0)	0.48 (0.14, 1.67)	0.250
Any acute kidney injury	36 (41.9)	70 (44.3)	0.90 (0.56, 1.44)	0.667
Acute kidney injury stage ≥ 2	21 (24.4)	38 (24.1)	1.02 (0.58, 1.80)	0.947
Acute kidney injury stage ≥ 3	7 (8.1)	30 (19.0)	0.38 (0.17, 0.86)	0.020
Newly-onset dialysis	4 (4.7)	21 (13.3)	0.32 (0.11, 0.90)	0.031
Delirium	19 (22.1)	29 (18.4)	1.26 (0.64, 2.50)	0.506
Post-operative atrial fibrillation	43 (50.0)	75 (47.5)	1.11 (0.67, 1.82)	0.690
Ventilator ≥ 2 days	62 (72.1)	117 (74.1)	0.91 (0.51, 1.63)	0.752
Ventilator ≥ 3 days	44 (51.2)	80 (50.6)	1.02 (0.63, 1.67)	0.933
Primary composite outcome	56 (65.1)	108 (68.4)	0.86 (0.50, 1.49)	0.602
**Continuous parameter**				
PRBC amount (U) in surgery	5.0 [3.0, 9.0]	4.0 [3.0, 8.0]	0.88 (− 0.52, 2.28)	0.215
PRBC amount (U) in hospitalization	6.0 [3.0, 10.0]	4.0 [3.0, 10.0]	0.41 (− 2.02, 2.84)	0.742
Ventilator (days)	3.0 [1.0, 5.0]	3.0 [1.0, 8.0]	− 1.94 (− 4.96, 1.07)	0.206
ICU duration (days)	4.0 [2.0, 7.0]	4.0 [2.0, 9.0]	− 1.17 (− 3.76, 1.43)	0.377
Hospital stays (days)	19.5 [13.0, 29.0]	18.0 [12.0, 29.0]	0.68 (− 6.47, 7.83)	0.852
Medical expenditure (USD × 10^3^)	23.4 ± 15.0	23.8 ± 16.9	− 0.37 (− 4.66, 3.93)	0.867

*B*, regression coefficient; OR, odds ratio; CI, confidence interval; ECMO, extracorporeal membrane oxygenation; PRBC, packed red blood cells; ICU, intensive care unit; USD, US dollars.

Data were presented as frequency (percentage) or mean ± standard deviation.

After propensity score matching, the dexmedetomidine group has a lower 30-day and in-hospital mortality rate than did the non-dexmedetomidine group (5.8% vs 13.9%; OR 0.39; 95% CI, 0.14–1.07; *p* = 0.067). The rate of the primary composite outcome, post-operative atrial fibrillation, delirium, respiratory failure, and stroke were similar between groups. The risk of AKIN stage 3 acute kidney injury was significantly lower in the dexmedetomidine group than in the non-dexmedetomidine group (8.1% vs 19.0%; OR 0.38; 95% CI 0.17–0.86; *p* = 0.020. The risk of newly-onset dialysis was also significantly lower in the dexmedetomidine group than in the non-dexmedetomidine group (4.7% vs 13.3%; OR 0.32; 95% CI 0.11–0.90; *p* = 0.031). But the risk of more than AKIN stage 1 or 2 acute kidney injury were similar between groups. The transfusion requirements including packed red blood cells during the peri-operative period or whole hospital course were similar. The Ventilator days, ICU stays, hospital stays, and medical expenditures were also similar.

## Discussion

In the analysis of consecutive patients undergoing type A aortic dissection at our multi-institutional hospital, after careful propensity score matching, in-hospital mortality was 5.8% in the dexmedetomidine group versus 12.8% in the non-dexmedetomidine group. There are several studies have studied the effect of dexmedetomidine on cardiac surgery including CABG or valve surgery^10,11^. To our knowledge, there is currently no study has evaluated the effects of dexmedetomidine on the outcome of aorta surgery.

The surgical mortality of type A aortic dissection lies a large part upon its initial presentation. One risk model proposed by IRAD investigators which including greater than 70 years, prior cardiac surgery, hypotension or shock at presentation, migrating pain, cardiac tamponade, any pulse deficit, and electrocardiogram with findings of myocardial ischemia or infarction^12^. We have carefully matched the above factors except for pulse deficit and electrocardiogram finding, however, residual confounding may have occurred due to unmatched or unknown confounders.

Acute kidney injury is a common complication after cardiac surgery. Studies suggest that dexmedetomidine might be protective. Cho and colleagues showed perioperative infusion of dexmedetomidine effectively reduced the incidence and severity of AKI^11^. Zhai and colleagues showed dexmedetomidine may attenuate the renal injury and decrease the incidence of AKI in patients undergoing cardiac valve replacement under CPB^13^. Peng and colleagues published a meta-analysis, including a total of 1308 patients. They concluded that perioperative dexmedetomidine administration provided protective effects against cardiac surgery-associated acute kidney injury^14^. Our data shows the risk of AKIN stage 3 acute kidney injury was significantly lower in the dexmedetomidine group and the risk of newly-onset dialysis was also significantly lower. Further randomized controlled trials is warranted.

Atrial fibrillation after cardiac surgery is common. Liu and colleagues showed dexmedetomidine sedation reduced the incidence of new-onset postoperative atrial fibrillation after cardiac surgery. However, their study only included 88 patients^15^. Zhu and colleagues report a meta-analysis of 1295 patients, which revealed that dexmedetomidine could not reduce the incidence of AF compared to control medicines following cardiac surgery^16^. The rate of postoperative atrial fibrillation in our study was 50.0% in the dexmedetomidine group and 47.5% in the non-dexmedetomidine group. The rate of postoperative atrial fibrillation seems higher than the general cardiac patient. Dexmedetomidine infusion does not affect the rate of postoperative atrial fibrillation in patients who received surgery for type A aortic dissection.

Dexmedetomidine has been evaluated for delirium not only in cardiac surgery patients but also in critical care patients and patients who received non-cardiac surgeries^17–19^.

A meta-analysis contains 969 patients in eight studies shows dexmedetomidine was associated with a lower risk of delirium (risk ratio, 0.40; 95% CI 0.24–0.64; *p* = 0.0002)^13^. Another meta-analysis included ten trials with 1387 patients shows perioperative dexmedetomidine administration decreased the incidence of delirium (risk ratio 0.46; 95% CI 0.34 to 0.62; *p* < 0.00001)^20^. The rate of delirium was similar between-group in our study. The reason could be related to circulatory arrest during surgery for acute type A aortic dissection, which is rarely needed in CABG or valve surgery.

ARDS is uncommon after general cardiac surgery but is common after surgery for type A aortic dissection. Su and colleagues showed 15.9% of patients had ARDS after surgical repair for type A aortic dissection^21^. In our study, after PSM, the dexmedetomidine group shows a lower rate of respiratory failure, albeit not significant.

### Limitation

This study had limitations. First, this is a retrospective database study, inherent limitations of observational studies remain, the potential confounding biases associated with a non-randomized study remain. Despite making adjustments for known confounders by using PSM, we could only investigate associations but could not infer causation. Residual confounding may have occurred due to unmeasured or unknown confounders. The distribution of these unmeasured confounders could differ substantially in the two groups, which may induce a biased estimate of the treatment effect. Second, the dexmedetomidine was reimbursed by NHI since 2007. There will be no selection bias regarding the economic issue. However, according to reimbursement condition, dexmedetomidine is limited to be used in a patient who is expected to be extubated shortly after the operation in patients who need sedation and pain relief within 24 h after surgery, and the use time should not exceed 24 h. Which could lead to a treatment bias that more stable patient was prescribed dexmedetomidine in the postoperative period. Third, the dexmedetomidine was given as infusion after operation instead of during operation or even after dissection before the operation. The anti-inflammatory effects of dexmedetomidine could be greater if the drug was infused during operation.

## Conclusions

This is the first study to evaluate the effect of perioperative dexmedetomidine use in patients who have received surgery for type A aortic dissection. Dexmedetomidine infusion after the operation was more likely to have a significantly lower risk of severe acute kidney injury and the need for hemodialysis. Further prospective randomized controlled trials should be constructed to confirm the clinical benefit of dexmedetomidine for patients after surgery for acute type A aortic dissection.

## Supplementary Information

Supplementary Information.
