# Supplementary material for: Effects of dexmedetomidine on surgery for type A acute aortic dissection outcome
Source: Sci Rep. 2022 Feb 17;12:2761. doi: 10.1038/s41598-022-06710-w (PMC8854389; doi:10.1038/s41598-022-06710-w)
Supplement: Supplementary file 1 — Supplementary Information. [file 41598_2022_6710_MOESM1_ESM.docx]

**Supplemental Table 1.** Baseline characteristics of the patients with and without acute kidney injury stage 3 (including dialysis) in the propensity score matched cohort

|  |  | Acute kidney injury stage 3 (including dialysis) | |  |
| --- | --- | --- | --- | --- |
| Variable | Available number | Yes (*n* = 37) | No (*n* = 207) | *P* |
| Demographics |  |  |  |  |
| Age, year | 244 | 56.9 ± 12.2 | 56.4 ± 13.7 | 0.829 |
| Male sex | 244 | 28 (75.7) | 134 (64.7) | 0.194 |
| BMI, kg/m^2^ | 223 | 29.1 ± 5.1 | 29.4 ± 23.8 | 0.954 |
| Previous cardiac surgery | 244 | 2 (5.4) | 1 (0.5) | 0.012 |
| Comorbidities |  |  |  |  |
| Hypertension | 244 | 24 (64.9) | 139 (67.1) | 0.786 |
| Coronary artery disease | 244 | 3 (8.1) | 12 (5.8) | 0.590 |
| Marfan syndrome | 244 | 2 (5.4) | 8 (3.9) | 0.663 |
| Diabetes mellitus | 244 | 3 (8.1) | 10 (4.8) | 0.414 |
| Chronic kidney disease | 244 | 7 (18.9) | 21 (10.1) | 0.123 |
| Liver disease | 244 | 4 (10.8) | 18 (8.7) | 0.679 |
| Atrial fibrillation | 244 | 3 (8.1) | 12 (5.8) | 0.590 |
| COPD | 244 | 0 (0.0) | 6 (2.9) | 0.294 |
| Old stroke | 244 | 3 (8.1) | 5 (2.4) | 0.073 |
| Pre-operative conditions |  |  |  |  |
| Tamponade or shock | 244 | 3 (8.1) | 18 (8.7) | 0.907 |
| Pre-operative lab data |  |  |  |  |
| Creatinine, mg/dL | 239 | 2.0 ± 2.1 | 1.2 ± 0.9 | <0.001 |
| WBC, 10^3^/uL | 241 | 13.3 ± 4.1 | 13.8 ± 5.2 | 0.548 |
| Platelet, 1000/uL | 241 | 193.2 ± 105.4 | 189.4 ± 62.9 | 0.765 |
| Hemoglobin, g/dL | 241 | 13.5 ± 2.2 | 13.5 ± 1.9 | 0.889 |
| BUN, mg/dL | 176 | 22.5 ± 14.1 | 18.4 ± 8.9 | 0.061 |
| Sodium, mg/dL | 239 | 138.3 ± 3.8 | 138.3 ± 3.1 | 0.979 |
| Potassium, mg/dL | 239 | 3.9 ± 0.6 | 3.7 ± 0.5 | 0.021 |
| Albumin, mg/dL | 69 | 3.3 ± 0.5 | 3.3 ± 0.6 | 0.644 |
| Lactic acid, mg/dL | 74 | 75.6 ± 34.6 | 52.3 ± 30.8 | 0.084 |
| HbA1c, % | 76 | 6.2 ± 0.6 | 6.0 ± 0.5 | 0.263 |
| AST, U/L | 165 | 39.0 [25.0, 64.0] | 36.0 [25.0, 70.0] | 0.144 |
| ALT, U/L | 178 | 28.0 [20.0, 50.0] | 24.0 [18.0, 34.0] | 0.803 |
| INR | 237 | 1.2 ± 0.1 | 1.1 ± 0.2 | 0.601 |
| Post-operative lab data |  |  |  |  |
| Platelet, 1000/uL | 244 | 134.8 ± 50.0 | 144.4 ± 42.1 | 0.217 |
| Hemoglobin, g/dL | 244 | 10.0 ± 2.2 | 10.7 ± 1.4 | 0.021 |
| Lactic acid, mg/dL | 161 | 96.6 ± 61.1 | 51.4 ± 28.0 | <0.001 |
| Proteinuria, mg | 72 | 100.0 [30.0, 100.0] | 30.0 [0.0, 30.0] | <0.001 |
| AST, U/L | 209 | 165.0 [82.5, 311.5] | 71.0 [51.0, 122.0] | <0.001 |
| ALT, U/L | 197 | 101.0 [34.0, 216.0] | 40.5 [24.5, 88.0] | 0.007 |
| SOFA score | 131 | 11.2 ± 1.9 | 10.5 ± 2.2 | 0.153 |
| Surgical data |  |  |  |  |
| Bypass time, min | 196 | 309.8 ± 98.3 | 241.3 ± 67.6 | <0.001 |
| Clamp time, min | 202 | 185.5 ± 65.2 | 156.5 ± 49.7 | 0.005 |
| Arrest time | 133 | 43.9 ± 27.2 | 44.0 ± 20.7 | 0.974 |
| Brain protection | 244 |  |  | 0.994 |
| Antegrade |  | 25 (67.6) | 140 (67.6) |  |
| Retrograde |  | 12 (32.4) | 67 (32.4) |  |
| Cerebral perfusion, min | 127 | 54.9 ± 30.1 | 48.1 ± 21.7 | 0.236 |
| Surgical extension |  |  |  |  |
| Partial or total aortic arch replacement | 244 | 8 (21.6) | 36 (17.4) | 0.538 |
| Aortic root replacement | 244 | 1 (2.7) | 8 (3.9) | 0.730 |
| Elephant trunk | 244 | 4 (10.8) | 16 (7.7) | 0.529 |
| Ascending aorta replacement only | 244 | 24 (64.9) | 147 (71.0) | 0.452 |

BMI, body mass index; COPD, chronic obstructive pulmonary disease; WBC, whole blood cell; BUN, blood urea nitrogen; HbA1c, glycated hemoglobin; AST, aspartate aminotransferase; ALT, alanine aminotransferase; SOFA, sequential organ failure assessment; INR, international normalized ratio;

Data were presented as frequency (percentage) or mean ± standard deviation or median [Quartile 1, Quartile 3].
